# Recycled Polypropylene/Strontium Ferrite Polymer Composite Materials with Electromagnetic Shielding Properties

**DOI:** 10.3390/polym16081129

**Published:** 2024-04-17

**Authors:** Alina Ruxandra Caramitu, Magdalena Valentina Lungu, Romeo Cristian Ciobanu, Ioana Ion, Mihai Marin, Virgil Marinescu, Jana Pintea, Sebastian Aradoaei, Oliver Daniel Schreiner

**Affiliations:** 1 National Institute for Research and Development in Electrical Engineering ICPE—CA Bucharest, 030138 Bucharest, Romania; alina.caramitu@icpe-ca.ro (A.R.C.); magdalena.lungu@icpe-ca.ro (M.V.L.); ioana.ion@icpe-ca.ro (I.I.); mihai.marin@icpe-ca.ro (M.M.); virgil.marinescu@icpe-ca.ro (V.M.); j_pintea@yahoo.com (J.P.); 2 Department of Electrical Measurements and Materials, Gheorghe Asachi Technical University, 700050 Iasi, Romania; sebastian-teodor.aradoaei@academic.tuiasi.ro (S.A.); oliver-daniel.schreiner@academic.tuiasi.ro (O.D.S.)

**Keywords:** polypropylene/strontium ferrite, PP/SrFe_12_O_19_, polymer composite, electromagnetic radiation, magnetic permeability, reflection shielding

## Abstract

This paper presents the obtaining and characterization of recycled polypropylene/strontium ferrite (PP/SrFe_12_O_19_) polymer composite materials with applications in the electromagnetic shielding of vehicle interiors (mainly automotive electronics—carcasses) from the electromagnetic radiation emitted mainly by exterior sources—electrical lines and supply sources—in terms of the development of the new electrical vehicles. With this aim, suitable polymer composite materials were developed using SrFe_12_O_19_ filler in two forms (powder and concentrate). The recycled PP polymer and composite materials with a PP/SrFe_12_O_19_ weight ratio of 75/25 and 70/30 were obtained in two stages, i.e., pellets by extrusion and samples for testing through a melt injection process. The characterization of the obtained materials took into account the requirements imposed by the desired applications. It consisted of determining the mechanical and dielectric properties, and microstructure analyses, along with the determination of the resistance to the action of a temperature of 70 °C, which is higher than the temperatures created during the summer inside vehicles. The performance of these materials as electromagnetic shields was assessed through functional tests consisting of the determination of magnetic permeability and the estimation of the electromagnetic shielding efficiency (SE). The obtained results confirmed the improvement of the mechanical, dielectric, and magnetic properties of the PP/SrFe_12_O_19_ composites compared to the selected PP polymers. It is also found that all the composite materials exhibited reflective shielding properties (SE_R_ from −71.5 dB to −56.7 dB), with very little absorption shielding. The most performant material was the composite made of PP/SrFe_12_O_19_ powder with a weight ratio of 70/30. The promising results recommend this composite material for potential use in automotive shielding applications against electromagnetic pollution.

## 1. Introduction

With recent advances in electromagnetic interference (EMI) shielding technologies and the rapid expansion of various electronic applications in the automotive industry, electromagnetic pollution has been steadily increasing. Electromagnetic waves can penetrate nearby electronic devices and adversely affect them or cause them to malfunction. With the increase in the number of electronically controlled automobile parts, accidents such as the malfunctioning and/or sudden acceleration of vehicles due to electromagnetic waves have become a major problem. Since electromagnetic waves can also harm people, the most effective EMI shielding technologies have demanded increased attention [1,2]. Electromagnetic radiation pollution can lead to damage to human health [2,3,4,5,6,7,8,9,10,11] and living matter [12,13,14,15,16], so it is urgently necessary to reduce it by researching and designing different new materials effective for this purpose. These materials must synergistically ensure a good attenuation of electromagnetic waves [17,18] and high resistance to the action of fungi [12,13,14,15,16,19,20], environmental conditions [21,22,23,24,25,26], mechanical stresses [27,28], excellent dielectric, magnetic and electromagnetic characteristics [29,30,31,32], and high resistance to the action of microwaves and UV radiation [33]. These conditions can be ensured through polymer-based composite materials, which have excellent mechanical properties and thermal stability, as well as high resistance to environmental conditions. The most used fillers for the realization of composite materials applied as protective shields for electromagnetic waves are metal powders (e.g., Cu, Ni, Ag, Al, Au, among others) [34], or ferrite powders such as (Zn, Fe, Ni, Mg, Cd)Fe_2_O_4_ with crystallite sizes below 30 nm, which present exceptional shielding properties [34,35,36]. For instance, the ferrite/polymer composite material developed by Radoń et al. [30] exhibited in the frequency range of 1.9–2.1 GHz a reflection shielding efficiency (RL) lower than −25 dB and a shielding effectiveness (SE) lower than −50 dB for a 0.8–1 cm thick layer.

Compared to spinel ferrites, hexagonal ferrites are also of significant interest as high-frequency microwave-absorbing materials due to their in-plane magnetic anisotropy and natural resonance in the GHz range. M-type strontium ferrites (SrFe_12_O_19_) and barium ferrites (Fe_12_BaO_19_) are typical examples of the hexagonal group that exhibit significant uniaxial anisotropy and strong saturation magnetization [37,38].

Some researchers [36] incorporated Mn- and Ti-doped strontium hexaferrite (SrFe_9_Mn_1.5_Ti_1.5_O_19_) powders with a magnetoplumbite structure into a polyvinylchloride (PVC) matrix in mass concentrations of 50%, 60%, 70%, and 80%. The PVC/ferrite polymer composites were obtained via hot pressing at 220 °C and 5.5 MPa in the form of discs with a diameter of 40 mm and a thickness of 1.8 mm. The composite filled with 70 wt.% ferrite showed a reflection loss below −15 dB in the frequency range of 16.4–19.4 GHz, and a good microwave absorption performance in the frequency range of 8–18 GHz.

Over the years, there has been research achieved to obtain composite materials using rubber as a polymer matrix and a compound ferrite of Cr and Mn as a filler material [31,39,40]. These polymer composite materials find their usefulness in the absorption of microwaves in the frequency range of 3–12 GHz. On the other hand, rubber magnetic composites containing Sr ferrite and Ba ferrite were obtained for use in manufacturing permanent magnets [39,41,42]. In another study [38], polymer composites made of 55 vol.% Sr-ferrite powder, 30 vol.% polypropylene (PP), and a certain polyethylene glycol (PEG) binder system were processed by the powder injection molding (PIM), then the binders were removed via extraction and thermal debinding, and the obtained samples were further sintered to prepare Sr-ferrite-based permanent magnets. Most of the research carried out so far on these composite materials has been focused on improving the performance of permanent magnets. On the other hand, ferrite polymer nanocomposites (NCPs) are of great interest as EMI shielding materials over a wide range of frequencies due to their good dielectric and mechanical properties, lightweight, and reduced cost [43].

This paper presents the obtaining and characterization of PP/SrFe_12_O_19_ polymer composite materials with applications in the electromagnetic shielding of vehicle interiors (mainly automotive electronics—carcasses) from the electromagnetic radiation emitted mainly by exterior sources—electrical lines and supply sources—in terms of the development of the new electrical vehicles. The PP polymer and composite materials with a PP/SrFe_12_O_19_ weight ratio of 75/25 and 70/30 were obtained in two stages, i.e., pellets via extrusion and samples for testing through a melt injection process. The characterization of the obtained materials consisted of carrying out specific tests to determine the physical, mechanical, dielectric, magnetic, and electromagnetic properties, as well as the resistance to the effect of temperature, and microstructure analysis, to select the most performant materials for the use as electromagnetic shields. The originality of our work consists of the selection of the polymer matrix and the filler, as well as the concentrations chosen to obtain the proposed composite materials. The purpose of this work was to obtain a lightweight polymer composite material with a reflection shielding efficiency (SE_R_) lower than −30 dB with applications against electromagnetic pollution.

## 2. Materials and Methods

### 2.1. Materials

The raw materials used to obtain the polymer composite materials are as follows:

- Polypropylene obtained from recycling from electronic waste [44] with the properties shown in Table 1. The use of recycled polypropylene is in line with the Directive 2000/53/EU on end-of-life vehicles.
- SrFe_12_O_19_ powder acquired from TODA Ferrite (Korea) with the properties shown in Table 2. One important reason for choosing strontium ferrites is that they are low-cost, widely used for ferrite permanent magnets, and may also become an important source of recycled matter in the near future.
- SrFe_12_O_19_ concentrate of F1 type acquired from Mate Co., Ltd. (Wake, Japan) with the trade name HM 1213-PA12 + 85% SrFe_12_O_19_ and the properties shown in Table 3.

### 2.2. Equipment and Methods

#### 2.2.1. Obtaining Polymer Composite Materials

Firstly, the component materials were homogenized through the mechanical mixing of the desired weight content of PP powder with SrFe_12_O_19_ powder/concentrate. The polymer composite materials were obtained in two stages, the first being composite pellets through extrusion, with the use of a laboratory twin screw extruder with PLC control type, Dongguan Baopin Precision Instruments Co., Ltd., Dongguan City, Guangdong Province (China), a length/diameter (L/D) ratio of 1:40, a screw rotation speed up to 120 rpm, frequency control, 5 heating zones, soft water circulating cooling, vibration feeding method, vacuum pomp, etc. Finally, the second stage includes samples for testing, via a melt injection process with a Dr. Boy 35A injection molding machine, Dr. Boy GmbH & Co. KG Neustadt-Fernthal, (Germany) with a screw diameter of 28 mm, a screw length/diameter (L/D) ratio of 18.6, a maximum clamping force of 350 kN, and a maximum working temperature of 300 °C. The concentrations of the obtained materials and their coding are presented in Table 4. Regarding the injection process, the working temperatures of 180–250 °C and clamping forces of 138–155 kN were used to achieve disc-shaped materials with a diameter of 30 ± 0.1 mm and a thickness of 2 ± 0.1 mm. 

#### 2.2.2. Material Characterization

##### Density Determination

The density of the M0-M4 materials was determined with Archimedes’ method in ethanol at 23 °C using an XS204 Mettler Toledo (Greifensee, Switzerland) hydrostatic balance equipped with a specific kit for solid materials, according to ASTM D792 standard [51]. Five specimens per material were measured and mean ± standard deviation (SD) values of the hydrostatic density were reported together with the calculated relative density and porosity values. The theoretical density of the composite materials was calculated with the rule of mixture.

##### Mechanical Characterization

The mechanical properties of the M0-M4 materials were investigated using a Micro-Combi Tester (MCT^2^) equipped with a diamond Berkovich indenter (CSM Instruments, Peseux, Switzerland) and load-controlled instrumented indentation testing (IIT), according to ISO 14577-1 standard [52]. The IIT measurements were performed with a maximum load (F_max_) of 400 mN in a quadratic loading, a time to loading/unloading F_max_ of 30 s, a hold time at F_max_ of 10 s, an approach speed of the indenter of 2000 nm/min, and an acquisition rate of 10 Hz. The indentation hardness (H_IT_), indentation elastic modulus (E_IT_), contact stiffness (S), indentation creep(C_IT_), elastic reverse and plastic deformation work of indentation (W_elast_ and W_plast_), and elastic part of indentation work (η_IT_) were determined via the Oliver and Pharr calculation method [53] as we detailed elsewhere [54] using a geometric factor (β) of 1.034 for the diamond Berkovich indenter with a Poisson’s ratio (ν_i_) of 0.07 and Young’s modulus (E_i_) of 1141 GPa, a Poisson’s ratio of 0.42 for the PP material (M0), and 0.37 for the polymer composite materials (M1-M4). Five measurements were realized per sample and mean ± standard deviation (SD) values were reported.

##### SEM Analyses

The microscopic structure and morphology investigation of the M0-M4 materials and filler materials (SrFe_12_O_19_ powder/concentrate) was performed with a field emission scanning electron microscope (FESEM), model Auriga (Carl Zeiss, Oberkochen, Germany), and a focused ion beam (FIB) column, model Canion (Orsay Physics, Fuveau, France). The SEM images were recorded on a cross-section of the solid samples and a charge compensation (CC) system, at an acceleration voltage of 5 kV with a secondary electron (SE) detector of Everhart Thornley type with a Faraday cup.

##### Thermal Analysis

The thermal analysis of the M0-M4 materials was conducted on an STA 449 F3 Jupiter thermal analyzer (Netzsch, Selb, Germany). The thermogravimetric (TG) and derivative thermogravimetric (DTG) curves of small amounts of material (10–15 mg) taken from each injection molded material and placed in 85 µL alumina (Al_2_O_3_) crucibles without lids were recorded in static air in the temperature range of 20–700 °C with a heating rate of 10 K/min.

##### Dielectric Characterization

The dielectric properties such as the real (ε′) and imaginary parts (ε″) of relative permittivity, along with the tangent of the dielectric loss angle (tg δ = ε″/ε′) of the M0-M4 samples were determined via dielectric spectroscopy using a Solartron 1260A dielectric spectrometer (Solartron Analytical, Farnborough, UK). The measurements were recorded using an electric field of an AC voltage amplitude of 3 V over a frequency range of 8–30 MHz and a measuring electrode with a diameter of 30 mm, according to the equations presented, e.g., in [55].

##### Temperature Resistance Testing

The resistance to the effect of temperature on the M0-M4 samples was carried out by analyzing the variation of tg δ and electrical resistivity for the estimation of the critical resistivity and time. The critical value of the electrical resistivity at which the material is considered degraded was chosen at the moment when the electrical resistivity decreased to 30% of the initial value. All samples were heated in a Memmert oven model UF 55, Memmert GmbH + Co. KG., Schwabach (Germany) at 70 °C for 72 h then cooled in air at room temperature to measure tg δ. This heating/cooling cycle was repeated 8 times. The selected temperature of 70 °C used in accelerated aging is higher than the temperature used inside vehicles.

##### Magnetic Characterization

The magnetic permeability of the M0-M4 samples was determined using a 4294A precision impedance analyzer (Agilent Technology Japan, Ltd., Tokyo, Japan) equipped with a 16454A Magnetic Material Test Fixture kit. Each sample was prepared as a hollow cylinder having an external diameter of 20 ± 0.1 mm, an internal diameter of 8 ± 0.1 mm, and a thickness of 2 ± 0.1 mm to measure the inductance at the ends of the wire. When the cylindrical sample is inserted into the Test Fixture kit, an ideal, single-turn inductor, with no flux leakage, is formed. Permeability is derived from the inductance of the core with the fixture. The real (μ′) and imaginary parts (μ″) of magnetic permeability were determined from the inductance measurements over the frequency range of 8–30 MHz, according to the following equations [56,57]:

(1) $$\mu^{\prime }=\frac{lL_{eff}}{\mu_{0}N^{2}A}$$

(2) $$\mu^{\prime \prime }=\frac{l(R_{eff}-R_{w})}{\mu_{0}N^{2}\omega A}$$

where *l* is the average length of the magnetic core, *L*_eff_ is the coil inductance, *R*_eff_ is the equivalent resistance of the magnetic core losses including the wire resistance, *N* is the number of windings, *R*_w_ is the wire resistance, *A* is the cross-sectional area of the magnetic core, ω is the angular frequency (ω = 2πf), f is the linear frequency (Hz), and µ_0_ is the permeability of vacuum (µ_0_ = 4π × 10^−7^ H/m).

## 3. Results and Discussion

### 3.1. Macrographic Aspect, Density, and Porosity

**Figure 1:**
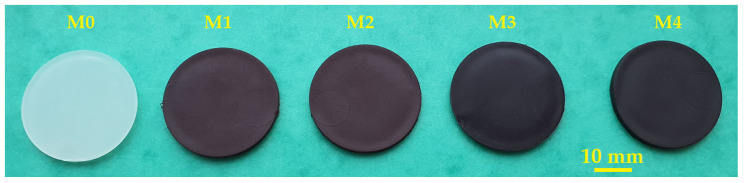
Macrographic aspect of the M0-M4 materials obtained through injection molding.

The macrographic aspect, along with the density and porosity of the M0-M4 materials obtained by injection molding is presented in Figure 1 and Table 5, respectively.

The results regarding the aspect (Figure 1) and density (Table 5) show the obtaining of uniform and homogeneous polymer composite materials (M1-M4) through the mechanical mixing of the desired weight content of PP powder with SrFe_12_O_19_ powder/concentrate, and injection molding. The used preparation methods are in line with other literature reports [43,58,59]. Additionally, Weidenfeller et al. [58] disclosed that the addition of 30 vol.% SrFe_12_O_19_ powders to a PP matrix had a benefic effect on reducing the cooling time during the injection-molding process of the ferrite polymer composite.

In our study, the obtained density of the polymer composite materials ranges between 1.068 ± 0.002 g/cm^3^ and 1.157 ± 0.002 g/cm^3^ and increased in the series: M3, M1, M4, and M2. The materials with SrFe_12_O_19_ powder filler exhibited higher density than the materials with SrFe_12_O_19_ filler concentrate at the same content of filler. It is also noticed that the addition of SrFe_12_O_19_ powder/concentrate filler increased by about 20–29% the density of the PP/SrFe_12_O_19_ polymer composites (M1-M4) compared to the density of the PP polymeric material (M0). However, all the developed composite materials (M1-M4) meet the lightweight requirement for use as EMI shielding materials.

### 3.2. Mechanical Properties

In contrast to conventional polymeric materials, the mechanical properties of polymer composite materials can be mostly controlled by the choice of components, the percentage and type of addition in the matrix (binder), the geometry and orientation of the fillers, and the preparation methods of mixtures and their processing techniques and conditions. The mechanical characteristics of polymer composite materials are strongly dependent on the mechanical strength, hardness, and stiffness of the fillers, as they are higher than those of the polymeric materials used as a matrix [60].

An instrumented indentation technique (IIT) such as nanoindentation with a Berkovitch diamond indenter has been utilized over the last twenty years for assessing the mechanical properties of polymer composite materials [61,62,63]. Furthermore, the calculation method employed in the IIT was introduced by Oliver and Pharr in 1992 [53]. It is widely used in data analysis for studying the mechanical behavior of solid surfaces, thin films, and coatings made of different materials [64].

**Figure 2:**
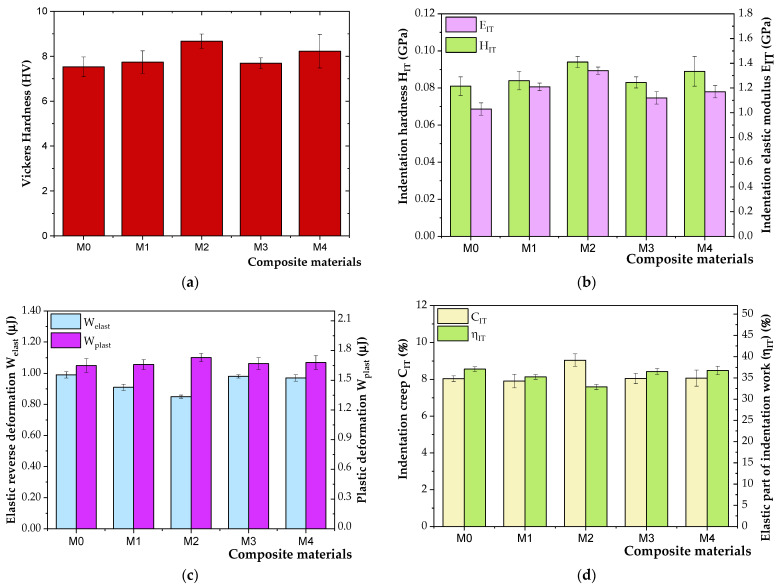
(**a**) Vickers hardness (HV); (**b**) indentation elastic modulus (E_IT_) and indentation hardness (H_IT_); (**c**) elastic reverse and plastic deformation (W_elast_, and W_plast_); and (**d**) indentation creep (C_IT_) and elastic part of indentation (η_IT_) of the studied materials.

Table 6 and Figure 2 present the experimental data obtained by nanoindentation and the Oliver and Pharr method for the studied composite materials.

In Table 6 and Figure 2, it is noticed that all the M1-M4 materials exhibited higher Vickers hardness HV ranging from 7.69 ± 0.24 to 8.67 ± 0.32, and better resistance to permanent (plastic) deformation or damage (H_IT_ between 0.083 ± 0.003 GPa and 0.094 ± 0.003 GPa) compared to the PP polymer (M0) (HV of 7.53 ± 0.44 and H_IT_ values of 0.081± 0.005 GPa). Moreover, the composite materials with the highest percentage in SrFe_12_O_19_ filler of 30 wt.% (M2 and M4) have the highest H_IT_ and HV hardness, while the lowest hardness is presented by the PP polymer without filler (M0). Therefore, it can be concluded that both H_IT_ and HV values increased for the M2 material by about 16% and 15%, respectively, by adding 30 wt.% SrFe_12_O_19_ powder filler in the PP matrix. The increase in the H_IT_ and HV values of the M4 material was about 11% and 9%, respectively, which is lower than for the M2 material due to the polyamide (PA) existent in the ferrite concentrate.

Regarding the indentation elastic modulus (E_IT_), which is similar to Young’s modulus, being determined from the slope of the unloading curve [65], it is found that the composite materials show higher E_IT_ (1.12 ± 0.03 GPa to 1.34 ± 0.03 GPa) than that of the PP polymer (1.03 ± 0.06 GPa). Additionally, the E_IT_ varies similarly to the trend of H_IT_ and HV. The E_IT_ results are in agreement with the results shown in the literature reports [66]. Polymers and polymer composites are generally found to have low E_IT_, indicating high polymer crystallinity. The more crystalline a polymer is (the content of crystalline areas is higher), the physical–mechanical properties improve but the fragility increases [57]. The increase in the elastic modulus of the polymer composites due to the filler addition can result from a good adhesion of the filler with the polymer matrix without the formation of large agglomerates, or from the filler interference on the polymer crystallization [60].

The IIT results show that the highest values of contact stiffness (S) were measured on the M2 material with the highest filler content (118.38 ± 1.83 mN/μm), followed by the M1 material (112.51 ± 1.92 mN/μm), these materials being obtained with 30 wt.% or 25 wt.% SrFe_12_O_19_ powder filler. The addition of 25–30 wt.% SrFe_12_O_19_ concentrate to the PP matrix also improved the contact stiffness at about 105 ± 5 mN/μm, while the lowest S values of 101.59 ± 3.80 mN/μm were reached in the PP polymer (M0). The creep behavior of the M0, M3, and M4 materials with PP and 25–30 wt.% PP/SrFe_12_O_19_ concentrate is similar. In contrast, the M1 material with 25 wt.% SrFe_12_O_19_ powder yielded the best creep resistance, while the increase in the SrFe_12_O_19_ powder content from 25 wt.% to 30 wt.% led to an increase in the C_IT_ values by about 14%, indicating a larger creep deformation [66]. Moreover, the M2 material exhibited higher plastic behavior (W_plast_ of 1.73 ± 0.04 μJ, and the plastic part of the indentation work of 67.11 ± 0.59%) than the other materials. All the M0-M1 materials exhibited a similar viscoelastic behavior showing higher W_plast_ values than the W_elast_ ones, but these values varied in a narrow range. The PP polymer (M0) exhibited the highest W_elast_ and η_IT_ values indicating higher elastic deformation ability due to preserving the polymer chains structure in case no filler is added to the polymer matrix.

### 3.3. SEM Analysis

SEM analysis was carried out for all the materials obtained through injection molding, as well as for the filler raw materials used to obtain them. All the SEM images were recorded on the cross-section of the solid samples and the charge compensation (CC) system. The employed CC system uses an injection of nitrogen gas on a locally analyzed surface, which allows for the obtaining of images without metal deposition on the surfaces of analyzed samples. Hence, the true state of the morphology of the surfaces of samples is highlighted without inducing the formation of any artifacts to the analyzed areas but decreases the quality of images (low smoothing of surfaces due to charge effect which is still present). It also can decrease the contrast of the obtained images. 

**Figure 3:**
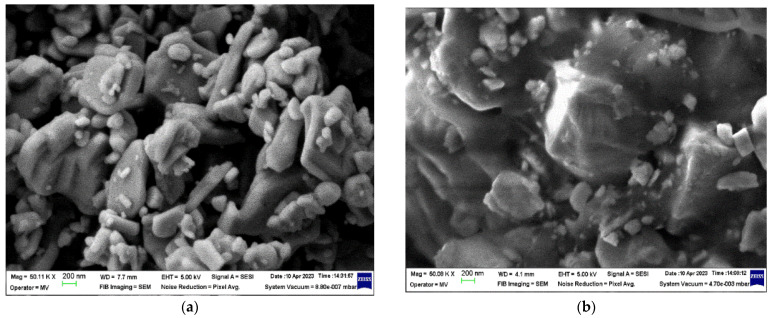
Microscopic images (50,000×) for (**a**) SrFe_12_O_19_ powder and (**b**) F1 concentrate.

**Figure 4:**
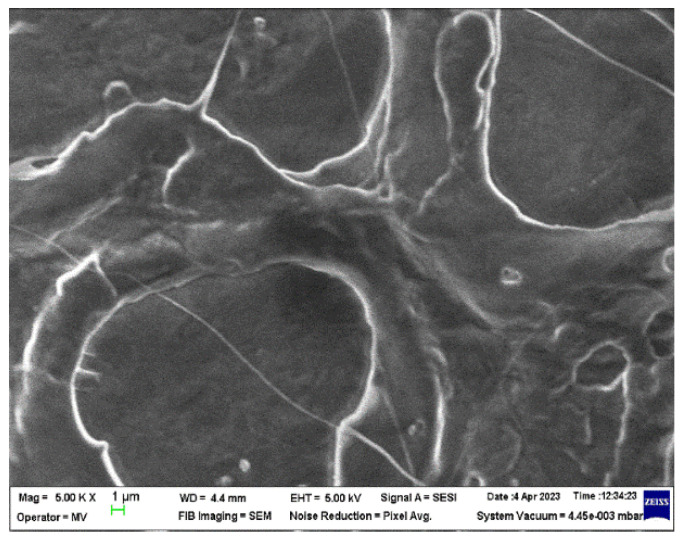
Microscopic image (5000×) for the PP polymeric material (M0) obtained through injection molding.

**Figure 5:**
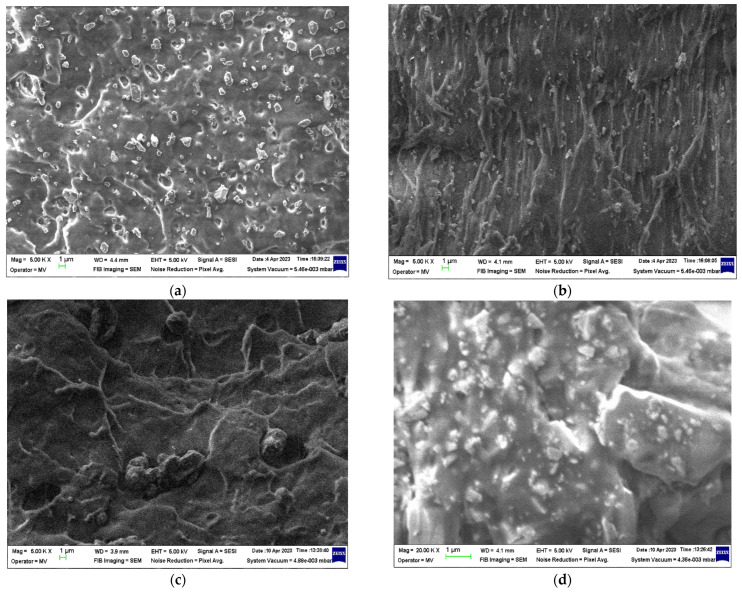
Microscopic images (5000× for (**a**–**c**) and 20,000× for (**d**)) for the PP/SrFe_12_O_19_ polymer composite materials: (**a**) M1, (**b**) M2, (**c**) M3, and (**d**) M4 obtained through injection molding.

The micrographs of the SrFe_12_O_19_ powder and F1 concentrate are presented in Figure 3, and the micrographs of the M0-M4 materials are presented in Figure 4 and Figure 5, respectively.

In the microscopic images of the ferrite fillers (Figure 3), a large grain size distribution of the SrFe_12_O_19_ particles in the micrometer range (<1 µm) can be seen for the ferrite powder. On the other hand, the grain size distribution of the SrFe_12_O_19_ particles incorporated in the F1-type ferrite concentrate cannot be differentiated in the polyamide (PA) matrix.

In the microscopic image of the M0 sample (Figure 4) the true state of the PP polymer can be observed as a blank surface only with an amorphous rheological morphology due to the injection molding process. The microscopic images of the M1-M4 samples (Figure 5) show different distribution behavior of SrFe_12_O_19_ filler in the PP polymer matrix.

After analyzing the micrographs performed on the composite materials (Figure 5), it can be observed that the M1 and M2 materials have higher homogeneity compared to the M3 and M4 materials with ferrite concentrate filler. This behavior is caused by the presence of the PA12 polymer existent in the filler concentrate, which has a higher melt flow index (Table 3) than the one of the PP polymer matrix (Table 1). The decrease in homogeneity is visible in the micrographs in the form of cluster-like agglomerates of ferrite particles in the PP polymer matrix.

### 3.4. Thermal Analysis

**Figure 6:**
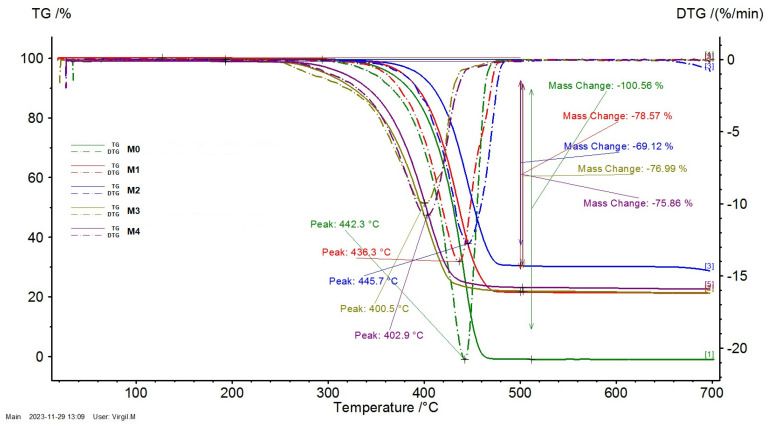
TG and DTG curves of the M0-M4 materials obtained through injection molding.

Figure 6 presents the TG and DTG curves of the M0-M4 materials obtained through injection molding.

The decomposition process of the PP polymer (M0) is at its maximum at 442.3 °C, while for the PP/SrFe_12_O_19_ polymer composite materials (M1-M4), this is dependent on the quantity and type of the utilized filler. 

The decomposition process of the composite materials with ferrite powder filler (M1 and M2) presents a maximum at 436.3 °C and 445.7 °C, respectively. On the other hand, the decomposition process of the materials with the F1-type ferrite concentrate filler (M3 and M4) presents a maximum at 400.5 °C and 402.9 °C, respectively. The decrease in the maximum of the decomposition process is determined by the presence of nitrogen atoms existent in the composition of the polyamide (PA) polymer.

In Figure 6, it can be noticed that after 450 °C, all the polymers are decomposed in the volatile matter and the resulting solid residue consists of ferrite filler. The mass loss is around 100% for the PP polymer (M0), whereas for the polymer composite materials (M1-M4), it ranges between 69% and 79%. The obtained mass loss values are in line with the designed content of the ferrite filler.

### 3.5. Dielectric Properties

**Figure 7:**
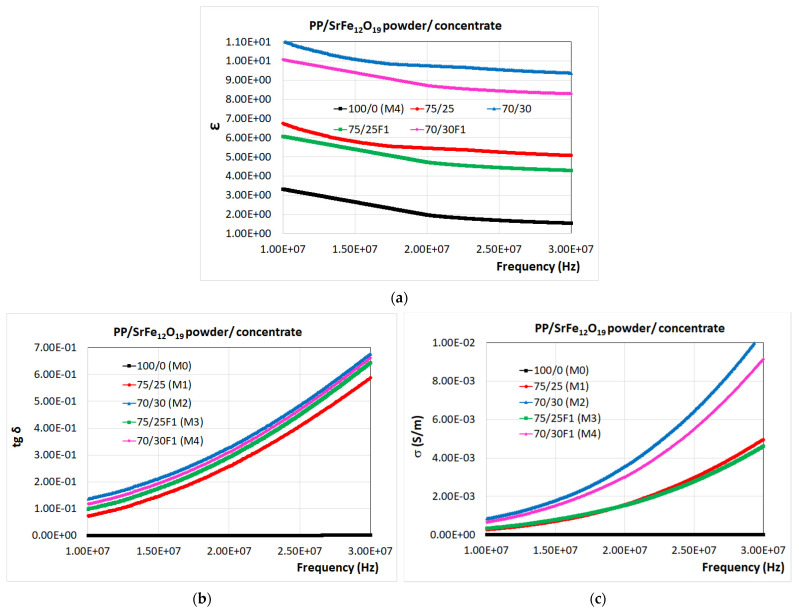
Variation in (**a**) relative dielectric permittivity (ε); (**b**) tangent of dielectric loss angle (tg δ); and (**c**) electrical conductivity (σ) for the studied composite materials (M1-M4) compared to the PP polymeric material (M0).

Figure 7 shows the variations of the tangent of the dielectric loss angle (tg δ) and electrical conductivity (σ) over the frequency range of 8–30 MHz for the studied composite materials (M1-M4) compared to the PP polymeric material (M0). The frequency interval was particularly chosen to respond to the need for electromagnetic shielding from the electromagnetic radiation emitted mainly by exterior sources—electrical lines and supply sources—in terms of the development of the new electrical vehicles [67], for which particular testing equipment is in the process of development [68].

When analyzing the experimental data obtained for ε, tg δ, and σ for the composite materials with fillers (M1-M4) comparatively with the PP polymer (Figure 7), it can be observed that the addition of SrFe_12_O_19_ powder/concentrate fillers leads to an increase mainly with regard to dielectric permittivity, but also with regard to both dielectric losses and electrical conductivities. Regarding the values of tg δ and σ for the recycled polypropylene (around 10^−4^ and 10^−9^, respectively), they are very low compared to the composite values, seen at the base of the graphics. 

Additionally, the ε, tg δ, and σ values of all the materials increased with the increase in frequency, in the series: M0, M1, M3, M4, and M2. The variation in the dielectric properties of polymer composites with frequency is typically ascribed to the dipole and interfacial polarization between the conductive filler and the polymer matrix, as well as to the relaxation mechanisms [34].

The M2 and M4 materials with the highest percentage (30 wt.%) in SrFe_12_O_19_ powder/concentrate show the greatest increases in electrical conductivity. Therefore, the obtained composite materials can be used as shielding materials since they are conductive and, thus, can contribute to the reflection mechanism of EMI shielding [34]. It is also noticed that the composite materials containing SrFe_12_O_19_ powder provide a better conductivity compared to that offered by the F1-type SrFe_12_O_19_ concentrate due to the achievement of homogeneous microstructure with uniform distribution of the filler particles into the polypropylene (PP) polymer matrix. The presence of the polyamide (PA) in the walls of the F1-type ferrite concentrate, a technology taken into account for the purpose of increasing the affinity of powders towards the PP matrix and diminishing flocculation effects, in fact determined the decrease in the electrical conductivity of the composite materials, since the PA walls are insulators with very low conductivity.

### 3.6. Temperature Resistance 

Temperature resistance testing was carried out by analyzing the variation in tg δ and electrical resistivity of all the samples subjected to accelerated aging (eight heating/cooling cycles) at 70 °C for 72 h/cycle and estimating, via calculations, the critical electrical resistivity and time at which the material can be considered degraded.

**Figure 8:**
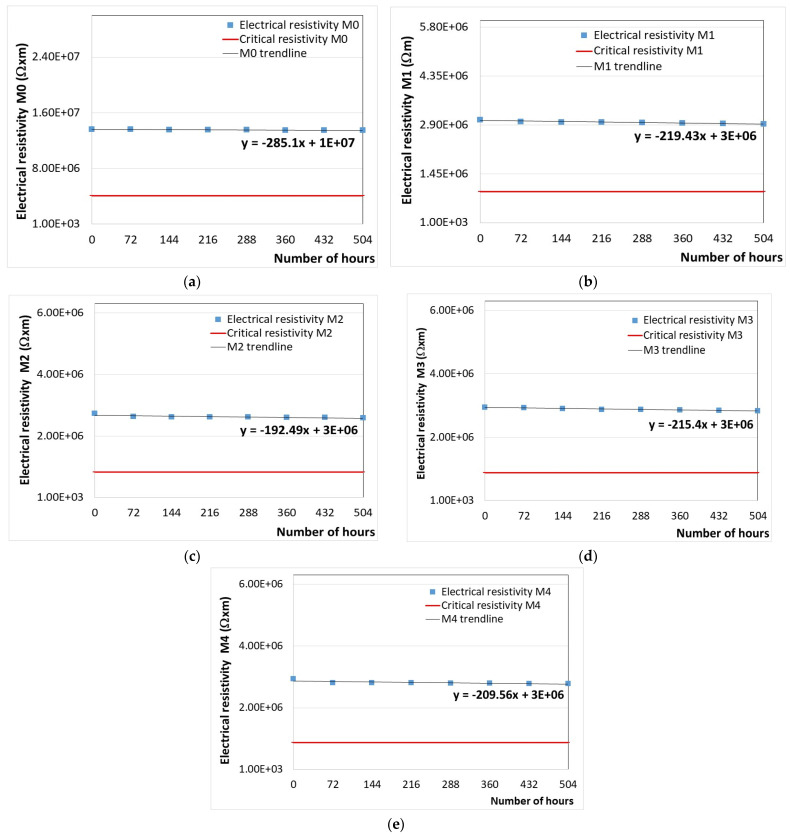
(**a**–**e**) Variation in the electrical resistivity of M0-M4 versus the exposure time at a temperature of 70 °C.

The variation in the electrical resistivity of the developed samples versus the exposure time at a temperature of 70 °C is presented in Figure 8, along with the first-degree equations (y = ax + b) that describe the trend of the variation curve.

Using first-degree equations for calculating the solutions of the equations given by each graph shown in Figure 8, we obtained the data from Table 7. Thus, the number of hours of thermal aging can be identified in a facile way. This information is very useful in industrial applications to replace, at an appropriate time, the PP polymer-based materials subjected to thermal degradation.

According to the results shown in Table 7, we can estimate the time for the replacement of the PP polymer and PP/SrFe_12_O_19_ composite materials before reaching their irreparable damage and the end of protection from electromagnetic radiation. Thus, it is found that the number of days after which the materials have to be replaced is 865 days for M0, 395 days for M1, 472 days for M2, 409 days for M3, and 422 days for M4.

It must be mentioned that the values achieved as above are related to the thermal exposure of materials according to the electrical engineering standard [69], i.e., related to the insulation class; in our case, they were assimilated to the lowest insulation class A for automotive electronics—carcasses, etc. (105 °C limit of use, 70 °C exposure to determine lifetime, and 40 °C maximum ambient temperature), herewith M2 and M4 exceeding 10,000 h for 70 °C exposure. Regarding the real lifetime for automotive applications, the maximum ambient temperature must be taken into account, i.e., 40 °C, and applying the Arrhenius equation for accelerated thermal tests, e.g., as described in [70], the real service values are obtained by extrapolating the data from Table 7, exceeding 10 years of normal usage for M2 and M4.

Comparing the composite materials with SrFe_12_O_19_ fillers from the point of view of the maintenance time of the electromagnetic shield characteristics, the following classification can be made: M2 > M4 > M3 > M1. However, the M0 material made of PP polymer exhibited the highest temperature resistance over those of the PP/SrFe_12_O_19_ composite materials. The reason for a lower value of lifetime for M4 compared to M2 may be related to the obtaining process of the composites in two stages, finalized by melt injection, when mutual interactions between the SrFe_12_O_19_ particles and polymer matrix can occur on the filler–polymer interface in the melt PP polymer/ferrite mixture, which may cause slight repulsive forces due to the different affinity of inorganic particles to polymer matrix, as described, e.g., in [31].

### 3.7. Magnetic Properties

**Figure 9:**
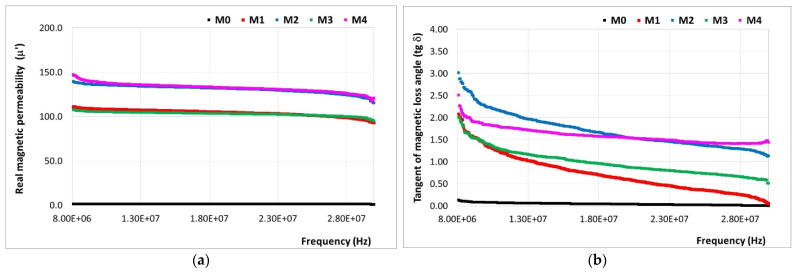
Variation in (**a**) real magnetic permeability (µ’) and (**b**) tangent of magnetic loss angle (tg δ) for the studied composite materials (M1-M4) compared to the PP polymeric material (M0).

The determination of the complex magnetic permeability of the developed materials was carried out in the range of 8–30 MHz. The real magnetic permeability (µ′) and tangent of the magnetic loss angle (tg δ = µ″/µ′) values are presented in Figure 9.

All the PP/SrFe_12_O_19_ polymer composite materials (M1-M4) exhibited superior values of the real magnetic permeability (μ′) than that of the PP polymer material (M0) over the studied frequency range. The μ′ values were highly influenced by the content and distribution of the magnetic SrFe_12_O_19_ filler in the PP polymer matrix. The M2 material with the highest content of ferrite powder filler (30 wt.%) and homogeneous structure (Figure 5) exhibited the highest real magnetic permeability. In the case of the M4 material prepared with 30 wt.% ferrite concentrate filler, the real magnetic permeability is less than that for M2, due to the presence of the polyamide (PA) polymer in the walls of the F1-type filler concentrate which has a lower magnetic permeability.

The tangent of magnetic loss angle was higher for the M4 material for frequencies exceeding 20 MHz, followed by the M2 material, while the other materials exhibited a lower comparative behavior over the whole frequency range.

On the other hand, it can be observed that the addition of SrFe_12_O_19_ powder into the PP polymer matrix does not lead to better magnetic loss along all frequency domains, as it was noticed for dielectric loss when compared to the addition of the SrFe_12_O_19_ concentrate. This difference is explained by the fact that, when taking into account the dialectic loss, the obtaining of composites using SrFe_12_O_19_ concentrate as a filler (M3 and M4), homogenization with the polypropylene (PP) polymer matrix is hampered by the polyamide (PA) existent in the F1-type ferrite concentrate, which is not fully miscible with polypropylene and may involve repulsive forces that may lead to the appearance of defects, resulting in a continuous decrease in dielectric parameters in all frequency domains, aspect negligible when speaking about magnetic parameters.

## 4. Conclusions

In this work, recycled polypropylene/strontium ferrite (PP/SrFe_12_O_19_) polymer composite materials were analyzed, with potential applications in electromagnetic shielding (mainly automotive electronics—carcasses) from the electromagnetic radiation emitted mainly by exterior sources—electrical lines and supply sources—in terms of the development of the new electrical vehicles. 

The recycled polypropylene was particularly analyzed, due to the unification of polymer matrices used/to be further used in automotive applications; hereby, polypropylene was appointed, and in terms of Directive 2000/53/EU on end-of-life vehicles [71], one of the requirements is that a minimum of 25% of the plastic used to build a new vehicle must be recycled. On the other hand, an important reason for choosing strontium ferrites is that they are low-cost, widely used for ferrite permanent magnets, and may also become an important source of recycled matter in the near future.

The composites were obtained in two stages, i.e., pellets by extrusion and samples for testing via a melt injection process and characterized comparatively with the PP polymer used as a matrix. The composite materials were prepared with SrFe_12_O_19_ filler in powder/concentrate form with mass concentrations expressed by the PP/SrFe_12_O_19_ ratio of 100/0, 75/25, and 70/30. The obtained materials were coded as M0 for the PP polymer, M1 and M2 for the materials with ferrite powder filler, and M3 and M4 for the materials with ferrite concentrate filler, with polyamide cover, a technology taken into account for the purpose of increasing the affinity of powders towards polypropylene matrix and diminishing flocculation effects. 

The characterization of the developed materials involved the determination of relevant physical, mechanical, dielectric, thermal, magnetic, and electromagnetic properties, as well as the study of the microstructure. 

The macrographic aspect and density values of (1.068–1.157) ± 0.002 g/cm^3^ show the obtaining of uniform, homogeneous, and lightweight PP/ferrite polymer composite materials (M1-M4) via the utilized preparation methods. The composite materials with SrFe_12_O_19_ powder filler exhibited higher density than the materials with SrFe_12_O_19_ filler concentrate at the same content of filler. The filler addition increased by about 20–29% the density of the M1-M4 materials compared to the density of the PP polymer (M0). 

The nanoindentation tests performed on the injection-molded materials disclosed an improvement in the mechanical properties due to the SrFe_12_O_19_ filler addition to the PP matrix. The indentation hardness (H_IT_) and Vickers hardness, as well as the indentation elastic modulus (E_IT_) and contact stiffness (S), increased with the filler content increase. 

The contribution of the SrFe_12_O_19_ powder filler toward the enhancement of the mechanical properties was higher than in the case of using the SrFe_12_O_19_ concentrate filler, which is a compound containing HM 1213—polyamide 12 (PA12) and 85% SrFe_12_O_19_ powder. The PP polymer (M0) exhibited the lowest hardness, elastic modulus, and contact stiffness. On the contrary, this material exhibited the highest W_elast_ and η_IT_ values indicating a higher elastic deformation ability due to preserving the polymer chain structure in case no filler is added to the polymer matrix. The creep behavior of the M0, M3, and M4 materials with PP and 25–30 wt.% PP/SrFe_12_O_19_ concentrate is similar, but the creep resistance was better for the composites with a lower content of SrFe_12_O_19_ filler. 

All the M0-M1 materials exhibited a similar viscoelastic behavior showing higher W_plast_ values than the W_elast_ ones. It was also noticed that the M2 material exhibited higher plastic behavior than the other materials. According to the results of the mechanical tests performed, the M2 composite material was chosen as the optimal variant.

The SEM analysis performed on the injection-molded materials revealed the true state of the PP polymeric material (M0) as a blank surface only with an amorphous rheological morphology, whereas the M1-M4 materials show different SrFe_12_O_19_ filler distribution behavior in the polypropylene (PP) polymer matrix. The M1 and M2 materials have higher homogeneity compared to the M3 and M4 materials with ferrite concentrate filler containing PA12 polymer with a higher melt flow index than the one of the PP polymer matrix. The decrease in the homogeneity of composite materials is visible in the micrographs as clusters-like agglomerates of ferrite particles in the PP polymer matrix.

The decomposition process of the PP polymer (M0) is at its maximum at 442.3 °C, while for the PP/SrFe_12_O_19_ polymer composite materials (M1-M4), this is dependent on the quantity and type of the utilized filler. The decomposition process of the M1 and M2 materials with ferrite powder filler presents a maximum at 436.3 °C and 445.7 °C, respectively. The decomposition process of the M3 and M4 materials with ferrite concentrate filler presents a maximum at 400.5 °C and 402.9 °C, respectively, caused by the presence of nitrogen atoms existent in the composition of the polyamide polymer. After 450 °C, all the polymers are decomposed in the volatile matter and the resulting solid residue consists of ferrite filler. The mass loss is around 100% for the PP polymer (M0) and in the range of 69–79% for the M1-M4 composite, which is in line with the designed content of the ferrite filler.

The dielectric tests performed on these composite materials over the frequency range of 8–30 MHz qualify the composite material coded with M2 as the material with the highest electrical conductivity (σ) among the analyzed materials. The addition of 30 wt.% SrFe_12_O_19_ powder into the PP matrix increased the conductivity of the M2 material compared to that of the M4 material made of PP and the same content of the F1-type ferrite concentrate. The decrease in electrical conductivity resulted from the presence of the polyamide (PA) in the walls of the ferrite concentrate. The PA walls are insulators with very low electrical conductivity which also induces in the M4 material a lower conductivity compared to that of the M2 material in which only polypropylene (PP) polymer appears.

The results obtained for the temperature resistance testing by investigating the thermal aging behavior of materials (eight heating/cooling cycles) at 70 °C for 72 h/cycle indicated the number of days after which the materials have to be replaced. The most temperature-resistant composite material was found to be the M2 material. The values achieved as above are related to the thermal exposure of materials according to the electrical engineering standard, i.e., related to the insulation class, in our case, they were assimilated to the lowest insulation class A for automotive electronics—carcasses, etc. (105 °C limit of use, 70 °C exposure to determine lifetime, and 40 °C maximum ambient temperature), herewith M2 and M4 exceeding 10,000 h for 70 °C exposure. Regarding the real lifetime for automotive applications, the maximum ambient temperature must be taken into account, i.e., 40 °C, and applying the Arrhenius equation for accelerated thermal tests, the real service values obtained through extrapolation exceeded 10 years of normal usage for M2 and M4. 

Regarding the obtained magnetic permeability and the magnetic loss, it can be concluded that both the M2 and M4 materials present high performance along the entire frequency field; however, M2 may be preferred, due to the fact that when obtaining composites using SrFe_12_O_19_ concentrate as a filler (M3 and M4), homogenization with the polypropylene (PP) polymer matrix is hampered by the polyamide (PA) existent in the F1-type ferrite concentrate, which is not fully miscible with polypropylene and may involve repulsive forces that may lead to the appearance of defects, resulting in a continuous decrease in dielectric parameters in all frequency domain, aspect negligible when speaking about magnetic parameters.

The promising results recommend the M2 polymer composite material for potential use in shielding applications against electromagnetic pollution, but further studies are necessary for the upscaling of the material in real-size applications, along with a precise analysis of the electromagnetic shielding effect.

## Figures and Tables

**Table 1:** Properties of polypropylene obtained from recycling from electronic waste.

Property Name	Standard	Property Value
Melt flow index (230 °C/2.16 kg)	ISO 1133 [45]	25 g/10 min
Density	ISO 1183 [46]	0.9 g/cm^3^
Vicat softening temperature (condition A120)	ISO 306 [47]	150 °C
Tensile stress at yield	ASTM D638 [48]	33 MPa
Charpy impact strength (23 °C/notched)	ISO 179 [49]	2.5 kJ/m^2^
Flexural modulus	ISO 178 [50]	1400 MPa

**Table 2:** Properties of SrFe_12_O_19_ powder.

Property Name	Property Value
Particle diameter	<1.05 μm
Molecular weight	1061.7 g/mol
Melting point	>450 °C (mL)
Density	5.18 g/mL at 25 °C (mL)
Solubility	Soluble in organic solvents

**Table 3:** Properties of F1-type SrFe_12_O_19_ concentrate.

Property Name	Property Value
Specific gravity	3.2 g/cm^3^
Melting point	190 °C
Residual magnetic field strength	235 mT
Coercive force	177 kA/m
Intrinsic coercive force	251 kA/m
Maximum stored energy	10.9 kJ/m^3^
Density after ripening	3.21 g/cm^3^
Melt flow index (270 °C/10 kg)	130 g/10 min
Bending strength	125 MPa
Impact strength	28 kJ/m^2^

**Table 4:** Material compositions and coding.

Material Code	Composition (wt.%) of PP/SrFe_12_O_19_ Powder	Composition (wt.%) of PP/SrFe_12_O_19_ Concentrate
M0	100/0	-
M1	75/25	-
M2	70/30	-
M3	-	75/25
M4	-	70/30

**Table 5:** Density and porosity of the M0-M4 materials obtained through injection molding.

Material Code	Theoretical Density (g/cm^3^)	Mean Density ± SD (g/cm^3^)	Relative Density (%)	Porosity (%)
M0	0.900	0.894 ± 0.002	99.33	0.67
M1	1.134	1.077 ± 0.002	94.95	5.05
M2	1.197	1.157 ± 0.002	96.69	3.31
M3	1.097	1.068 ± 0.002	97.32	2.68
M4	1.148	1.138 ± 0.019	99.15	0.85

**Table 6:** Mean ± SD values of the mechanical properties (indentation hardness (H_IT_), indentation elastic modulus (E_IT_), contact stiffness (S), indentation creep (C_IT_), elastic reverse deformation work of indentation (W_elast_), plastic deformation work of indentation (W_plast_), and elastic part of indentation work (η_IT_)) of the materials determined through nanoindentation and the Oliver and Pharr method.

Material Code	H_IT_ (GPa)	Vickers Hardness HV	E_IT_ (GPa)	S (mN/μm)	C_IT_ (%)	W_elast_ (μJ)	W_plast_ (μJ)	η_IT_ (%)
M0	0.081 ± 0.005	7.53 ± 0.44	1.03 ± 0.06	101.59 ± 3.80	8.04 ± 0.15	0.99 ± 0.02	1.65 ± 0.07	37.07 ± 0.57
M1	0.084 ± 0.005	7.74 ± 0.50	1.21 ± 0.05	112.51 ± 1.92	7.91 ± 0.37	0.91 ± 0.02	1.66 ± 0.05	35.25 ± 0.55
M2	0.094 ± 0.003	8.67 ± 0.32	1.34 ± 0.03	118.38 ± 1.83	9.04 ± 0.34	0.85 ± 0.01	1.73 ± 0.04	32.89 ± 0.59
M3	0.083 ± 0.003	7.69 ± 0.24	1.12 ± 0.03	104.71 ± 5.27	8.05 ± 0.28	0.98 ± 0.01	1.70 ± 0.06	36.52 ± 0.71
M4	0.089 ± 0.008	8.23 ± 0.75	1.17 ± 0.05	105.43 ± 2.88	8.07 ± 0.44	0.97 ± 0.02	1.66 ± 0.07	36.75 ± 1.02

**Table 7:** Results for determining the critical electrical resistivity and time at which the developed materials can be considered degraded after thermal aging at 70 °C.

Material Code	Equation	Critical Electrical Resistivity (Ω·m)	Critical Time (h)	Critical Time (days)
M0	y = −285.1x + 1 × 10^7^	4,084,298	20,750	865
M1	y = −219.43x + 3 × 10^6^	917,492	9491	395
M2	y = −192.49x + 3 × 10^6^	820,393	11,323	472
M3	y = −215.4x + 3 × 10^6^	884,210	9823	409
M4	y = −209.56x + 3 × 10^6^	879,685	10,118	422

## Data Availability

Data are contained within the article.
